# Consequences of the Novel ALS-Associated *KIF5A* Variant c.2993-6C > A for Exon 27 Splicing and Axonal Transport of SFPQ

**DOI:** 10.1212/NXG.0000000000200362

**Published:** 2026-03-11

**Authors:** Guy A. Rouleau, Ziqi Yu, Jay P. Ross, Daniel Rochefort, Boting Li, Kate Bornais, Marvin Chum, Sali M.K. Farhan, Patrick A. Dion

**Affiliations:** 1 Montreal Neurological Institute-Hospital, QC, Canada;; 2 Department of Neurology and Neurosurgery, McGill University, Montréal, QC, Canada;; 3 Department of Human Genetics, McGill University, Montréal, QC, Canada;; 4 Division of Experimental Medicine, Faculty of Medicine and Health Sciences, McGill University, Montréal, QC, Canada; and; 5 Division of Neurology, Department of Medicine, McMaster University, Hamilton, ON, Canada.

## Abstract

**Background and Objectives:**

Recent studies have identified variants in the kinesin family member 5A (*KIF5A*) gene that predispose to amyotrophic lateral sclerosis (ALS). These ALS-linked *KIF5A* variants lead to the exclusion of exon 27, resulting in the production of a mutated protein with an altered C-terminal region (KIF5A ΔExon27). Through whole genome sequencing, we identified a novel *KIF5A* intronic variant, rs1057522322 (c.2993-6C > A; chr12:57582596C > A, GRCh38.p14), in a family segregating ALS. Our goal is to investigate the effect of this variant on exon 27 splicing and to assess its functional consequences on KIF5A-mediated cargo transport.

**Methods:**

Induced pluripotent stem cells (iPSCs) were generated from siblings with and without the c.2993-6C > A variant. RT-PCR was performed on RNA extracted from iPSC-derived neurons to assess exon 27 splicing. Functional studies were conducted on iPSC-derived motor neurons (MNs).

**Results:**

RT-PCR confirmed that the c.2993-6C > A variant induced exon 27 skipping in *KIF5A*. Immunofluorescent staining showed that KIF5A ΔExon27 abolished the axonal interaction with splicing factor proline- and glutamine-rich, a cargo specifically transported by KIF5A. Under stress conditions, MNs carrying the c.2993-6C > A variant exhibited TDP-43 proteinopathy.

**Discussion:**

*KIF5A* intronic variant c.2993-6C > A could be a risk factor for ALS. KIF5A ΔExon27 impairs KIF5A-mediated cargo transport and contributes to ALS pathogenesis in a TDP-43–dependent manner.

## Introduction

Amyotrophic lateral sclerosis (ALS) is a fatal neurodegenerative disorder characterized by the progressive loss of upper and lower motor neurons (MNs), leading to muscle weakness, atrophy, and eventual paralysis.^1^ The average survival time for individuals diagnosed with ALS is typically between 2 and 5 years, and no effective treatments currently exist.^1^ While most ALS cases are sporadic, approximately 10% are associated with specific pathogenic genetic variants.^2^ The past decade has seen significant advances in understanding ALS genetics. Since the identification of *SOD1* as the first well-established genetic risk factor for ALS in 1993, variants in over 30 additional genes, including *TARDBP*, *FUS*, and *C9orf72*, have been implicated in the disease.^3,4^ More recently, genome-wide association studies (GWAS) have revealed variants in the kinesin family member 5A (*KIF5A*) gene as a genetic risk factor responsible for ALS.^5,6^

Kinesins, also known as kinesin superfamily proteins (KIFs), are ATP-dependent motor proteins that play key roles in mitosis and the intracellular transport of cargo along microtubules (MTs).^7^ Kinesin-1 (or conventional kinesin, KIF5) is a founding member of KIFs.^7^ Kinesin-1 motor is essential for neuronal development and maintenance of neuronal integrity by facilitating the anterograde axonal transport of various cargoes, including organelles, vesicles, and protein complexes.^8^ Kinesin-1 is a heterotetramer consisting of 2 kinesin heavy chains (KHCs) and 2 kinesin light chains (KLCs).^9^ While the heavy chains are responsible for motor activity and ATP hydrolysis, the light chains function as adapter molecules, facilitating cargo binding to kinesins.^9^ KHC has 3 isoforms: KIF5A, KIF5B, and KIF5C. While KIF5B is ubiquitously expressed across various cell types, KIF5A and KIF5C are specifically expressed in neurons.^10^ Structurally, KHC consists of an N-terminal motor domain that binds to MTs in an ATP-dependent manner, a central coiled-coil stalk domain that mediates KHC dimerization, and a C-terminal domain that regulates cargo binding through interactions with KLC and other adaptor proteins.^7^ Notably, most ALS-associated *KIF5A* variants are clustered in the 5′ and 3′ splice junctions of exon 27 within its C-terminal tail.^5,6^ Normally, exon 27 is included in mature RNA. However, ALS-linked *KIF5A* splice junction variants are known to cause a complete skipping of exon 27, which encodes amino acids 998–1,007.^5,6^ This exon skipping results in a *KIF5A* transcript with a frameshift at amino acid 998, leading to the deletion of the 34 amino acids and the addition of 39 aberrant amino acids to the C-terminal cargo binding domain of KIF5A.^5,6^

Disrupted RNA metabolism is one of the major pathogenesis pathways in ALS.^1^ Splicing factor proline- and glutamine-rich (SFPQ) is a ubiquitously expressed RNA binding protein that is critical in neuronal development and has been implicated in several neurodegenerative diseases, including ALS.^11^ In addition to being genetically linked to ALS, SFPQ dysregulation, such as intron retention and reduced expression, has emerged as a novel pathologic feature common across patients with ALS.^12,13^ Studies have found that SFPQ interacts specifically with the KIF5A heavy chain in association with its cargo adaptor KLC1.^14,15^ The trafficking of SFPQ-RNA granules to the distal ends of axons by KIF5A/KLC1 motor is essential for the development and maintenance of MN axons.^14,15^

In this study, we identified a novel *KIF5A* intronic variant, rs1057522322 (c.2993-6C > A; chr12:57582596C > A, GRCh38.p14), in a family with a history of ALS. We demonstrated its potential pathogenic role in ALS by showing the presence of a skipped exon 27 splice isoform of *KIF5A*. We explored the functional consequences of the resulting KIF5A ΔExon27 protein by assessing its ability to transport of SFPQ, a cargo specifically transported by KIF5A. We also investigated whether TDP-43 proteinopathy is associated with the ALS-linked *KIF5A* variants. Our findings suggest that KIF5A ΔExon27 disrupts SFPQ trafficking, and TDP-43 is involved in the pathogenesis of *KIF5A*-related ALS.

## Methods

### Gene Sequencing

Whole genome sequencing (WGS) was performed on the proband as previously described.^16^ Briefly, DNA was prepared from whole blood using standard salting out procedures. Genes known to harbor variants imparting risk for ALS at the time of study conception were assessed for rare (minor allele frequency <0.001 in the gnomAD v3.1.2 population frequency database) protein or splice-site altering variants. The clinical testing was performed at a diagnostic laboratory namely, Athena Diagnostics, and the following genes were tested with no clinically relevant variants reports including any variants of uncertain significance, likely pathogenic or pathogenic variants: *ALS2*, *ANG*, *ANXA11*, *ARHGEF28*, *CFAP410*, *CCNF*, *CHCHD10*, *DAO*, *DCTN1*, *DNAJC7*, *FIG4*, *FUS*, *HNRNPA1*, *MAPT*, *MATR3*, *MOBP*, *NEFH*, *NEK1*, *OPTN*, *PFN1*, *PNPLA6*, *PRPH*, *SCFD1*, *SETX*, *SIGMAR1*, *SOD1*, *SQSTM1*, *TAF15*, *TARDBP*, *TBK1*, *TIA1*, *TUBA4A*, *UBQLN2*, *UNC13A*, *VAPB*, *VCP*, *GRN*, and *MAPT*. Testing for the known pathogenic hexanucleotide repeat expansion in *C9orf72* was conducted via conventional repeat-primed PCR as previously described^17^ with no pathogenic repeats identified. *KIF5A* variant validation was performed through Sanger sequencing. The sequencing was performed using the following primers.

c.2993-6>A Fwd—5′ TGT​AGC​TTG​GGA​TAA​CTA​AGG​AG 3′
c.2993-6>A Rev—5′ CAC​AGA​TGG​GAT​TGG​AGG​AG 3′

### iPSC Culture

Peripheral blood mononuclear cells (PBMCs) were reprogrammed into induced pluripotent stem cells (iPSCs) using the CytoTune iPS Sendai reprogramming kit (Thermo Fisher Scientific) according to the manufacture's protocol. iPSCs were cultured on a Matrigel (Corning)-coated plate with mTeSR1 medium (STEMCELL Technologies) at 37°C under 5% CO_2_ in a 100% humidity incubator. Cells were passaged at approximately 70%–80% confluency.

### Neural Progenitor Cells Differentiation

Neural progenitor cells (NPCs) were induced from iPSCs with STEMdiff SMADi Neural Induction kit according to the manufacturer's monolayer culture protocol. Briefly, a single-cell suspension of iPSC was generated using Gentle Cell Dissociation Reagent (STEMCELL Technologies). 2 × 10^6^ cells/well were seeded onto a single well of a poly-l-ornithine hydrobromide (PLO, Sigma Aldrich) and Laminin (Thermo Fisher Scientific)-coated 6-well plate and cultured in the induction medium composed of STEMdiff Neural Induction Medium + SMADi and 10-μM Y-27632. Daily full medium change was performed with the induction medium until the cells reached 80%–90% confluency. The cells were passaged using Accutase (STEMCELL Technologies) and were cultured as described above. The cells were passaged twice before maintaining in STEMdiff Neural Progenitor Medium until experimental use.

### MN Differentiation

For the final differentiation into MNs, a previously published protocol was modified for our studies.^18^ Briefly, NPCs were dissociated with Accutase and seeded at a density of 0.7 × 10^5^ cells/coverslip on a PLO/laminin-coated 24-well plate in MN induction medium. The induction medium consists of a 1:1 mix of KO-DMEM:F12 (Gibco) and Neurobasal Medium (Gibco) supplemented with 1× antibiotic-antimycotic (Gibco), 1× B27 (Thermo Fisher Scientific), 1× N2 (Thermo Fisher Scientific), 1× nonessential amino acids (NEAA, Gibco), 1× GlutaMAX (Thermo Fisher Scientific), 0.1-mM L-ascorbic acid (STEMCELL Technologies), 10-μM all-trans retinoic acid (Sigma Aldrich), 100-ng/mL recombinant SHH (STEMCELL Technologies), 1-μM purmorphamine (Abcam), and 1-µM SAG dihydrochloride (Sigma Aldrich). Daily medium change was performed for 6 days. The cells were then maintained in the MN maturation medium, composed of 1:1 KO-DMEM:F12 and Neurobasal Medium supplemented with 1× antibiotic-antimycotic, 1× B27, 1× N2, 1× NEAA, 1× GlutaMAX, 0.1-mM L-ascorbic acid, 10-ng/mL CNTF (Gibco), 10-ng/mL BDNF (Gibco), 10-ng/mL NT-3 (STEMCELL Technologies), and 10-ng/mL GDNF (STEMCELL Technologies), until neurons are ready to use. Maturation media were changed 3 times a week.

### RT-PCR Analysis

Total RNA was prepared from iPSC-derived NPCs using Trizol reagent. Reverse transcription was performed using SuperScript VILO cDNA Synthesis Kit in a 20-µL reaction volume according to the manufacturer's protocol. RT-PCR was carried out in a 20-µL reaction volume using GoTag Flexi DNA polymerase (Promega), 2-µL room temperature (RT) reaction (representing 50 ng input of RNA), and forward and reverse primer (0.5 µM). Amplification conditions were as follows: 95°C for 2 (95°C for 20s, 55°C for 20s, 72°C for) × 35 cycles, followed by an extension stage of 72°C for 2 minutes and a 4°C hold. Amplification of the normal splice forms was performed using the primers F1 (CAG​TGG​AGC​CAC​ATC​TTC​TG) and R1 (TCT​CTT​GGT​GGA​GAG​GGA​AA). For the amplification of the variant splice form, primer F2 (CCA​ACA​TGG​ACA​ATG​GAG​TGA), which spans exons 26 and 28, was used in combination with R1. The band intensities were quantified using ImageLab.

### Cell Viability Assay

iPSC-derived MNs were kept in culture until day 30. After fixation in 5% sucrose/3.7% formaldehyde in PBS, cells were incubated with DAPI (1:5,000, Sigma D9542) and imaged using a Leica SP8 confocal microscope. Cell numbers were visually counted using ImageJ cell counter.

### Arsenite Treatment

iPSC-derived MNs were treated with 0.5-mM sodium arsenite (Sigma-Aldrich) for 90 minutes to replicate stress conditions. After treatment, cells were washed once with PBS before fixing with 5% sucrose/3.7% formaldehyde in PBS for 1 hour at room RT.

### Immunocytochemistry and Confocal Imaging

All procedures were conducted at RT unless otherwise specified. Cells were washed twice with PBS and fixed with 5% sucrose/3.7% formaldehyde in PBS for 1 hour. After fixation, neurons were permeabilized using the same buffer containing 0.2% Triton × 100 for 2 minutes. Then, neurons were washed twice with PBS and incubated in 50-mM NH_4_CL in PBS for 10 minutes. Neurons were rinsed twice in PBS before blocking with 10% of Donkey Serum (DS) in PBS for 20 minutes. Neurons were incubated with the primary antibodies diluted in 5% DS/0.05% Triton × 100 in PBS overnight at 4°C. Neurons were washed 3 times with PBS for 5 minutes. The following primary antibodies were used: rabbit anti-Nestin (1:200; N5413), mouse anti-SOX2 (1:200; AB79351), mouse anti-Tubulin β-III (1:200; T8660), rabbit anti-Tubulin β-III (1:1,000; 802,001), chicken anti-Microtubule Associated Protein 2 (1:1,000; MBS502140), rabbit anti-Olig-2 (1:200; AB9610), rabbit anti-HB9 (1:200; ABN174), goat anti-Choline Acetyltransferase (1:200; AB144P), mouse anti-Neurofilament H (clone SMI-32; 1:200; 801,701), rabbit anti-Kinesin 5A (1:500, NBP2-57903), mouse anti-SFPQ (1:500, 67,129-1-lg), rabbit anti-TDP43 (1:200; 10782-2-AP), mouse anti-TDP-43-phospho-Ser409/410 (1:1,000, TIP-PTD-M01A), and mouse anti-TIAR (1:100; 610,352). Neurons were then incubated with appropriate secondary antibodies conjugated to Alexa 488, 555, or 647 (1:1,000) diluted in 5% DS/0.05% Triton × 100 in PBS for 1 hour. Neurons were washed 3 times with PBS for 5 minutes. DAPI (1:5,000, Sigma D9542) was used to label the nuclei (10 minutes). Neurons were washed 3 times with PBS and once in ddH_2_O before mounting with ProLong Diamond Antifade Mountant (Thermo Fisher Scientific). The images were acquired using a Leica SP8 confocal microscope and analyzed with ImageJ. The colocalization of SFPQ and KIF5A was quantified using ComDet ImageJ plugin, and the colocalization of TDP-43 with the nucleus was quantified using Colocalization Colormap ImageJ plugin.

### Statistical Analysis

For all experiments, data are represented as mean ± SD or SEM of 3 biological replicates. Statistics were performed using the two-tailed homoscedastic Student's *t* test. The threshold for significance was set at *p* ≤ 0.05.

### Standard Protocol Approvals, Registrations, and Patient Consents

All the experiments using human materials were approved by McGill University Health Centre Research Ethics Board (IRB00010120). Whole blood or PBMCs of consenting individuals were collected after obtaining written informed consent in accordance with the institutional human ethics committee guidelines.

### Data Availability

We declare that we take full responsibility for the data, the analyses and interpretation, and the conduct of the research; that we have full access to all of the data; and that we have the right to publish any and all data, separate and apart from the guidance of any sponsor.

## Results

### Identification of Novel *KIF5A* Variant in an ALS Family

**Figure 1:**
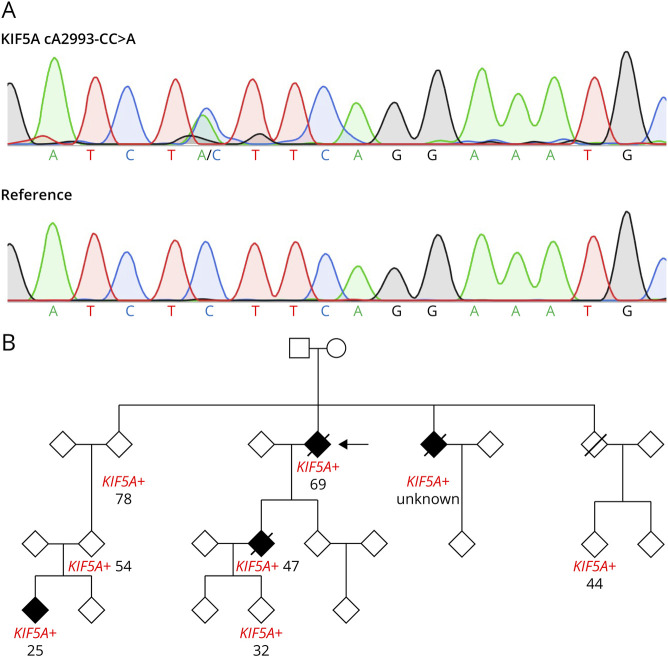
*KIF5A* Variant and Pedigree of the Family With the c.2993-C>A Variant (A) Sanger sequence chromatograms of the *KIF5A* c.2993-6C > A variant identified in the ALS family. The heterozygous single nucleotide variant is clearly demonstrated. (B) The pedigree structure of the ALS family with the *KIF5A* c.2993-6C > A variant. For ethical reasons, we anonymized the sex. The age at onset or the age at sample collection is shown below each individual (y/o). Open symbol: unaffected; open symbol with notation of KIF5A+: carrier; filled symbol: affected; diagonal line: deceased; square: male; circle: female; diamond: unknown sex; arrow: proband.

The proband of a multigeneration pedigree was subjected to WGS after inconclusive clinical genetic testing. After sequencing quality control and ruling out the presence of variants in other ALS genes, we identified a heterozygous *KIF5A* exon 27–5′ splice junction variant, rs1057522322 (c.2993-6C > A; chr12:57582596C > A, GRCh38.p14). This variant was located within intron 26, 6 nucleotides upstream of the exon 27, and was previously classified as an intronic variant of unknown significance (VUS) according to the American College of Medical Genetics and Genomics criteria.^19^ To validate the association of this variant with ALS, we performed Sanger sequencing on the abbreviated pedigree. The c.2993-6C > A variant was detected in the proband and other affected relatives but was absent in unaffected family members (Figure 1, A and B), suggesting that c.2993-6C > A could be a plausible pathogenic candidate responsible for the disease phenotype. While the variant is carried by all affected individuals in the pedigree, it is also present in unaffected individuals (Figure 1B), indicating an incomplete penetrance of this variant. In addition, the age at onset among affected individuals and the ages of unaffected carriers at the time of sampling show substantial variability (Figure 1B), suggesting that additional genetic or epigenetic modifiers may influence disease penetrance and age at onset.

### Intronic Variant c.2993-6C > A Alters KIF5A Exon 27 Splicing

**Figure 2:**
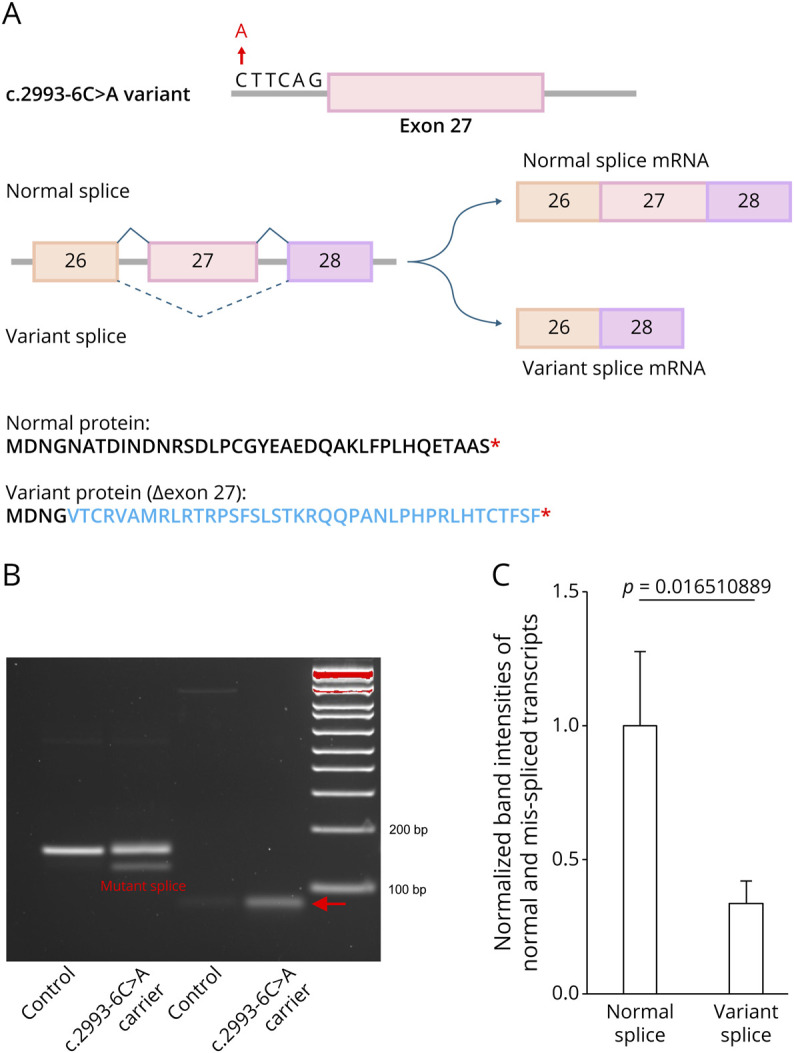
*KIF5A* 5′ Splice Junction Variant c.2993-6C>A Causes Exon 27 Skipping (A) ALS-associated *KIF5A* single nucleotide variants could induce skipping of exon 27 and result in an aberrant mRNA transcript, leading to an extended disrupted C-terminal peptide sequence. The amino acids in blue indicate the divergence from the normal KIF5A protein. (B) RT-PCR was performed using RNA derived from iPSC-derived NPCs from participants with and without (control) the variant c.2993-6C > A using primers to amplify either both reference (155 bp) and variant (127 bp) splice forms (lane 1 and 2) or specifically the variant splice form (80 bp, lane 3 and 4). The arrow indicates the position of the variant specific splice form. (C) Quantifications of the normal splicing and mis-splicing transcripts in culture, with the band intensities normalized to the normal splicing product (mean ± SD, n = 3). iPSC = induced pluripotent stem cell; NPC = neural progenitor cell.

ALS-linked *KIF5A* splice junction variants are known to cause a complete skipping of exon 27, resulting in the exon 26 spliced directly to exon 28, generating a variant protein with an altered C-terminal region^5,6^ (Figure 2A). Thus, we hypothesized that the intronic variant c.2993-6C > A might cause a skipping of exon 27 in *KIF5A*. We first used SpliceAI to predict the potential splicing effect of this variant; however, the prediction yielded a low score of 0.22, suggesting it was unlikely to have a strong effect. Because *KIF5A* is primarily expressed in neuronal cells, we reprogrammed PBMCs sampled from 2 siblings, 1 with and 1 without the single nucleotide variant c.2993-6C > A into iPSCs to generate human neurons. To study if this 5′ splice junction variant might have an effect on exon 27 splicing, we extracted RNA from iPSC-derived NPCs and performed RT-PCR analysis to test for the presence of exon 27 (eFigure 1). Our results demonstrated that the c.2993-6C > A variant induces exon 27 skipping, producing a transcript with a skipped exon that was absent in the control line, with an approximate ratio of 3:1 for normal splice to variant splice transcripts (Figure 2, B and C). Furthermore, our experimental evidence refutes the low effect prediction made by SpliceAI, highlighting the limitations of relying solely on computational predictions without empirical validation. In the RT-PCR experiment using primers designed to specifically amplify the variant splice transcript, the expected band of 80bp is present in the variant line (Figure 2B). However, a faint band is also detectable in the control line (Figure 2B), indicating that exon 27 exclusion occurs at low levels at baseline, consistent with the GTEx RNA-seq data from healthy brain tissues (eFigure 2).

### Variant KIF5A Fails to Colocalize With SFPQ in iPSC-Derived MNs

**Figure 3:**
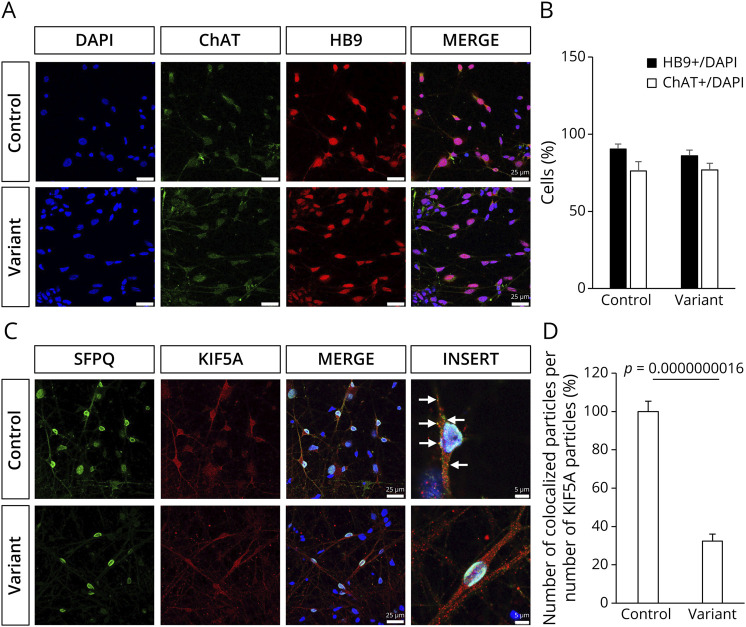
Variant KIF5A Protein Impairs SFPQ Transport in iPSC-Derived MNs (A) KIF5A control and variant iPSC line differentiated into MNs at 15 days in culture, displaying the MNs marker, ChAT (green), and HB9 (red). Scale bar = 25 µm. (B) Differentiation efficiency of KIF5A MNs (mean ± SD, n = 3). (C) Representative images of the colocalization (yellow, indicated by white arrows) of SFPQ (green) and KIF5A (red) in iPSC-derived MNs. Dapi (blue) was used to label the nuclei. Scale bar = 25 µm or 5 µm. (D) Quantification of the colocalized particles, with the number of colocalized particles normalized to the total number of KIF5A particles in each image. The resulting values were further normalized to those observed in the control cell line (mean ± SEM, n = 3). iPSC = induced pluripotent stem cell; MN = motor neuron; SFPQ = splicing factor proline- and glutamine-rich.

SFPQ is a cargo specially transported by the KIF5A/KLC1 motor complex, and studies have shown that KIF5A ΔExon27 can still interact with KLC1.^14,20^ We then aimed to investigate the ability of KIF5A ΔExon27 to transport SFPQ in iPSC-derived MNs. After 15 days in culture, the cells were positive for MN markers HB9 and ChAT, with similar differentiation efficiency between the 2 lines (Figure 3, A and B). Similarly, both cell lines showed comparable differentiation efficiency when co-immunolabelled for OLIG2 and TUJ1 (eFigure 3, A and B). Given that ALS is a late-onset neurodegenerative disorder, we extended the MN culture to 30 days to allow the MNs to become more functionally active^18^ and stained them with SMI-32, another well-established marker for MNs (eFigure 3C). Cell viability assays of these 30-day-old iPSC-derived MNs showed that the c.2993-6C > A variant did not affect MN survival (eFigure 3D). Next, we performed immunofluorescent staining to assess the colocalization of KIF5A and SFPQ. Because most commercially available antibodies against KIF5A recognize the C-terminal tail of KIF5A, which is affected by the ALS-associated KIF5A variants, we used an antibody targeting the stalk domain (spanning amino acids 535 to 585) of KIF5A. Our data showed that while SFPQ interacts efficiently with reference KIF5A in the axons, cells expressing KIF5A ΔExon27 protein show impaired axonal interactions with SFPQ, despite the presence of a functional reference copy of KIF5A (Figure 3E).

### TDP-43 Localization Is Not Affected by *KIF5A* Variant Under Physiologic Conditions

**Figure 4:**
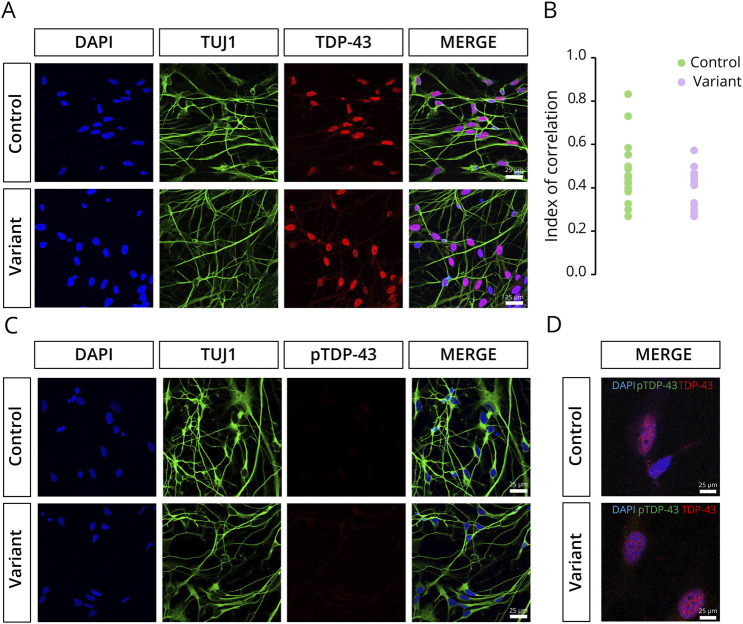
TDP-43 Proteinopathy Is Absent in Variant iPSC-Derived MNs Under Physiologic Conditions (A) TDP-43 (red) is predominantly localized within the nucleus (blue) in both control and variant lines, with neurons labeled by the neuronal marker TUJ1 (green). Scale bar = 25 µm. (B) Nuclear localization of TDP-43 is quantified by the correlation index, where higher scores indicate strong nuclear colocalization (n = 3). (C) No cytoplasmic phosphorylated TDP-43 (pTDP-43) inclusions were detected in either the control or variant lines (n = 3). Scale bar = 25 µm. (D) Enlarged view of double labeling of TDP-43 (red) and pTDP-43 (green) (n = 3). Scale bar = 25 µm. iPSC = induced pluripotent stem cell; MN = motor neuron.

Because cytoplasmic aggregation of TDP-43 is seen at autopsy in 97% of all patients with ALS,^1^ we sought to determine whether TDP-43 positive inclusions spontaneously form in MNs carrying the KIF5A variant. In both control KIF5A and KIF5A ΔExon27-expressing cells, TDP-43 remained primarily localized in the nucleus (Figure 4, A and B). In ALS, phosphorylation of TDP-43 at serine 403/404 and 409/410 (pTDP-43) is commonly observed in deposited TDP-43 inclusions.^21^ To further study the potential involvement of TDP-43 proteinopathy in ALS-associated *KIF5A* variants, we examined the presence of cytoplasmic pTDP-43 aggregates. Consistent with our TDP-43 localization data, no cytoplasmic pTDP-43–positive inclusions were detected in the KIF5A ΔExon27-expressing cells under physiologic conditions (Figure 4, C and D).

### TDP-43 Pathology Is Triggered by Acute Oxidative Stress in Variant MNs

**Figure 5:**
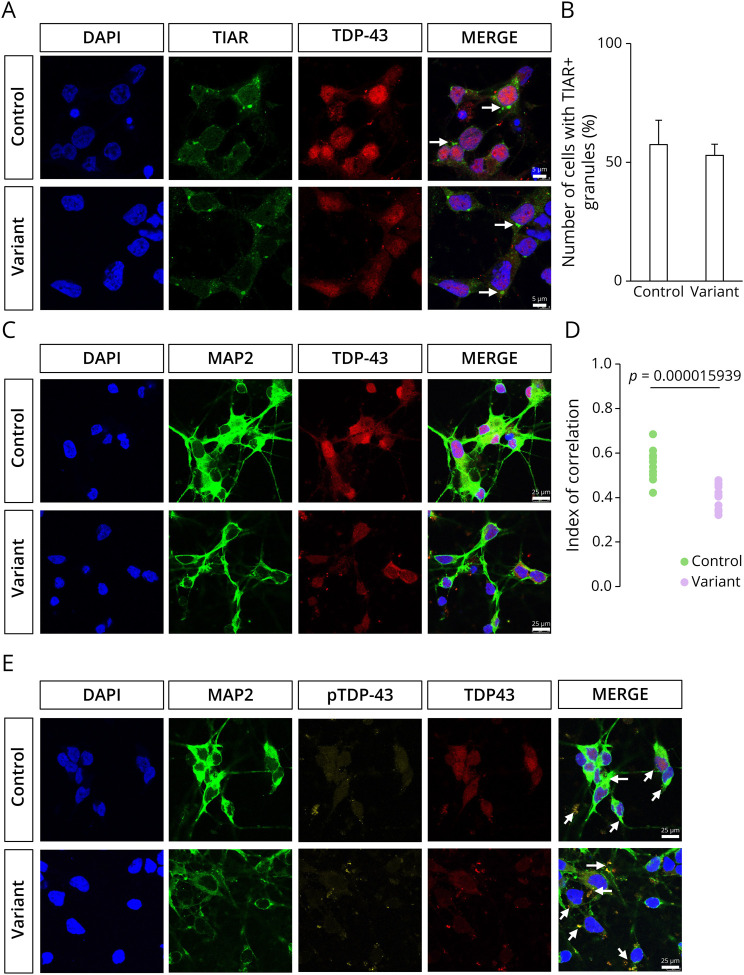
Acute Oxidative Stress Induces TDP-43 Proteinopathy in Variant iPSC-Derived MNs (A) TIAR-positive stress granules (green, indicated by white arrows) are formed after sodium arsenite treatment. Scale bar = 5 µm. (B) Quantitative analysis of numbers of stress granules (%) on sodium arsenite treatment in control and variant MNs (mean ± SD, n = 3). (C) TDP-43 (red) is mislocalized to the cytoplasm after sodium arsenite treatment, with neurons labeled by neuronal marker MAP2 (green). Scale bar = 25 µm. (D) Nuclear localization of TDP-43 is quantified by the correlation index, where higher scores indicate strong nuclear colocalization (n = 3). (E) Cytoplasmic pTDP-43– and TDP-43–positive inclusions were detected in MNs, as indicated by the white arrows (n = 3). Scale bar = 25 µm. iPSC = induced pluripotent stem cell; MN = motor neuron.

Several studies using iPSC-derived ALS disease models have reported an absence of TDP-43 pathology, even in iPSC-derived MNs carrying TDP-43 variants.^22,23^ TDP-43 pathology is only seen under stress conditions. We tested whether oxidative stress could induce TDP-43 pathology in MNs carrying *KIF5A* variants. To this end, we exposed MNs to 0.5-mM sodium arsenite for 90 minutes, as previously described in the literature.^23-25^ Because TDP-43 can modulate G3BP1 protein levels, we used TIAR as an alternative stress granule marker to detect the formation of stress granules.^26^ In untreated MNs, TIAR was diffusely distributed in the nucleus and cytoplasm in both control and variant lines (eFigure 4). After acute oxidative stress, TIAR-positive granules formed in both control and variant MNs, with TDP-43 being recruited to only a few of the TIAR-positive granules (Figure 5, A and B). Furthermore, we found that TDP-43 expression was reduced in the nuclei and became mislocalized to the cytoplasm in variant MNs, whereas in control MNs, despite some cytoplasmic mislocalization, TDP-43 remained predominantly nuclear (Figure 5, C and D). To further assess TDP-43 proteinopathy, we examined cytoplasmic pTDP-43 levels. Under stress conditions, pTDP-43 expression was increased in both control and variant MNs, whereas its expression level was barely detectable under physiologic conditions (Figures 4C and 5E). In addition, pTDP-43 exhibited a granular and/or fibrillar cytoplasmic distribution, with variant MNs displaying a higher number and larger size of pTDP-43 puncta compared with control MNs (Figure 5E).

## Discussion

Advances in GWAS and next-generation sequencing techniques have allowed the discovery of ALS-associated genes to be conducted in large sample sets.^27^ In 2018, a GWAS identified *KIF5A* as an ALS gene.^5,6^ Subsequent studies have reported additional patients with ALS carrying *KIF5A* variants.^28-30^ The *KIF5A* splice junction variant rs1057522322 (c.2993-6C > A) that we found in our family had been previously interpreted as a VUS. Our results suggest that the c.2993-6C > A variant causes exon 27 skipping in *KIF5A* and shows incomplete penetrance for ALS. We also provide evidence supporting an autosomal-dominant inheritance pattern in this ALS family. Among familial ALS cases caused by inherited gene variants, penetrance varies substantially depending on the gene and the specific variant within it. For instance, reported estimates of maximum population penetrance for the 4 most common ALS genes are approximately 33% for *C9orf72*, 54% for *SOD1*, 38% for *TARDBP*, and 19% for *FUS.*^3,4,31^ In *KIF5A*-associated ALS, exon 27 skipping represents a major molecular alteration. Notably, this exon exclusion has also been observed at a very low level both in our control iPSC-derived neurons and in healthy brain tissues, whereas the presence of ALS-linked *KIF5A* variants markedly increase the proportion of exon 27 skipping. Therefore, genetic and/or epigenetic mechanisms regulating one's ability to suppress this splicing shift and thus limiting exon 27 exclusion may determine disease penetrance, age at onset, and progression. Accordingly, future therapeutic strategies for *KIF5A*-associated ALS should focus on targeting the reduction of exon 27 skipping as a mean to prevent the downstream deleterious cascade.

The primary function of KIF5A is moving intracellular cargo toward the plus-end of the axons. For instance, it has been shown that SFPQ interacts selectively to KIF5A/KLC1 motors, and its trafficking to the distal end of the axon is key for promoting axonal survival.^14^ ALS-associated *KIF5A* variants disrupt the cargo binding domain of the protein, which could affect its binding to SFPQ. Hence, we investigated whether KIF5A ΔExon27 retains its ability to bind and transport SFPQ in iPSC-derived MNs. To our surprise, our results indicated that the variant KIF5A protein almost completely abolished the axonal transport of SFPQ in iPSC-derived MNs. Studies have found that homozygous *Kif5a* knockout in mice is embryonic lethal, whereas heterozygous mice are normal, suggesting that having 1 functional copy of the *KIF5A* gene is sufficient for maintaining normal physiologic functions in mice.^32^ This could be attributed to the fact that KIF5A functions as dimer.^9^ Because the ALS-linked *KIF5A* variants do not affect the region where KIF5A dimerizes, the variant KIF5A protein is likely dimerizing with the reference copy. Thus, the resulting variant-reference KIF5A dimer might be structurally defective, hindering its ability to bind to SFPQ. In fact, it has been shown that KIF5A ΔExon27 interacts with reference KIF5A and is neurotoxic.^33-35^ In line with our data, the variant KIF5A shows impaired interactions with mitochondria, which are a well-known cargo of KIF5A.^33,34,36^ An altered distribution of mitochondria has also been reported in an in vivo ALS-KIF5A drosophila model expressing human KIF5A ΔExon27, further supporting a global axonal transport defect caused by KIF5A ΔExon27.^37^ Another potential explanation is that the variant KIF5A protein may competitively inhibit proper cargo binding of reference KIF5A by restricting the functional KIF5A motors available for anterograde transport. KIF5A ΔExon27 can sequester reference KIF5A into oligomers, which are found to be more active than KIF5A dimers.^20,33^ This is because, in cells, KIF5A exists in an autoinhibited state with its C-terminal tail binding to the N-terminal motor domain in the absence of cargo to prevent the rapid depletion of ATP.^38,39^ However, several studies have revealed that KIF5A ΔExon27 induces hyperactivation of KIF5A, which promotes KIF5A motor self-association, causing a drastically increased processivity on MTs.^20,33,35,37^ This hyperactive state of variant KIF5A, together with the ability of KIF5A ΔExon27 to sequester reference KIF5A into aggregates, may cause a rapid depletion of cytoplasmic reference KIF5A available for proper transport of cargoes, resulting in the observed defects in SFPQ trafficking. In addition, it has been reported that KIF5A ΔExon27 has a faster turnover rate compared with reference KIF5A.^34^ This may also limit the number of reference KIF5A available for anterograde transport as it may alter the clearance of KIF5A ΔExon27-reference KIF5A heterodimer, causing an inability to recycle reference KIF5A to the cytoplasm.

The Y-acidic motif in SFPQ enables its binding to KIF5A/KLC1 motors.^14^ Similarly, a loss of axonal pool of SFPQ has been observed in MNs carrying ALS-associated SFPQ variants, N533H and L534I, which are adjacent to the Y-acidic motif of SFPQ.^40^ Variants in the N-terminal region of KIF5A have been linked to Charcot-Marie-Tooth (CMT) disease type 2.^41^ Of interest, a CMT2-associated KIF5A^R280H^ variant, which resides within the motor domain of KIF5A, has been found to reduce SFPQ binding by approximately 50% compared with reference KIF5A.^14^ This suggests that SFPQ binding to KIF5A is highly sensitive to structural changes in KIF5A, with variants in the cargo binding domain having a more profound effect than those in the motor domain. Overall, our findings indicate that the interaction between SFPQ and KIF5A may be impaired in *KIF5A*-related ALS, potentially representing an important downstream effect of *KIF5A* variants in ALS.

A major pathologic hallmark that is seen in ∼97% of patients with ALS is the depletion of nuclear TDP-43 and the buildup of cytoplasmic TDP-43 aggregates.^1^ Under physiologic conditions, we did not detect aberrant cytoplasmic TDP-43 mislocalization or aberrant expression of pTDP-43 in both control and variant MNs. Consistent with our findings, TDP-43 has been found within the nucleus in HEK293 cells expressing either reference KIF5A or KIF5A ΔExon27.^33^ However, on exposure to stress, TDP-43 mislocalized to the cytoplasm and formed cytoplasmic puncta, which were also positive for pTDP-43 in control and variant MNs, but with variant MNs exhibiting a more pronounced effect. Furthermore, we did not observe significant recruitment of TDP-43 into stress granules with exposure to acute oxidative stress, consistent with previous reports.^23^ Notably, KIF5A is predisposed to form oligomers in cells, and several studies on ALS-associated *KIF5A* variants have reported the formation of cytoplasmic KIF5A aggregates in cells.^20,33,34,37,42^ Combining our data, we propose the hypothesis that ALS-linked *KIF5A* variants may initially exert toxicity through a TDP-43 independent mechanism, possibly by forming aggregates on their own. Similar to other cytoplasmic inclusions seen in patients with ALS, these KIF5A aggregates may sequester other proteins and translationally active mRNA involved in protein quality control, making cells more vulnerable to stress and thereby facilitating TDP-43 mislocalization and aggregation.^43^ When stress persists and becomes chronic, as often seen in neurodegeneration, the protein quality control system eventually collapses, at which stage TDP-43 plays a central role in driving cellular toxicity, leading to ALS phenotypes. Furthermore, our data support the notion that genetic predisposition alone is insufficient for ALS pathogenesis and that a second hit, such as aging or environmental factors, is needed for disease initiation.

There are limitations in our study. First, we did not distinguish between reference and variant KIF5A in our functional analyses. Because of the limited availability of commercially available KIF5A antibodies, it was challenging to study the specific contributions of reference KIF5A and the KIF5A ΔExon27 protein in axonal transport of SFPQ. Moreover, because most of the commercially available antibodies recognize the mutated regions of KIF5A, we were unable to perform co-immunoprecipitation to assess the direct interaction between KIF5A dimers and SFPQ. The absence of colocalization of KIF5A and SFPQ in variant cell line does not necessarily reflect a complete inability of reference KIF5A to interact with SFPQ in the presence of KIF5A ΔExon27, because the interaction may be lost only on reference and variant KIF5A oligomerization. With appropriate antibodies, future studies could investigate the interactions between SFPQ and various KIF5A dimer configurations, specifically, reference-reference, reference-variant, and variant-variant dimers. Finally, while we demonstrated a disruption in SFPQ trafficking in cells expressing variant KIF5A, we did not investigate the downstream effects of this loss of axonal SFPQ in the variant line. Given the transport of SFPQ-RNA granules is important for the maintenance of axon in neurons,^14,15^ future studies could explore whether the impaired axonal transport of SFPQ caused by variant KIF5A would lead to axonal degeneration in MNs, which may provide further insight into the broader mechanisms of KIF5A dysfunction in ALS.

Defects in axonal transport have been widely recognized as a hallmark of various neurodegenerative disorders. We demonstrated that the *KIF5A* c.2993-6C > A variant impairs the function of iPSC-derived MNs, which further highlights the critical role of cytoskeletal dynamics in the pathogenesis of ALS. Our findings suggest that ALS-associated KIF5A variants lead to disrupted interactions of KIF5A with SFPQ. In addition, we show that ALS-associated *KIF5A* variant leads to TDP-43 pathology in iPSC-derived MNs on exposure to stress. Collectively, our study provides new insights into the synergic pathogenic mechanism of axonal transport defects and disrupted RNA metabolism in ALS.

## Glossary

ALS: amyotrophic lateral sclerosis
ANG: Angiogenin
ATP: Adenosine triphosphate
BDNF: Brain-Derived Neurotrophic Factor
CMT: Charcot-Marie Tooth
CNTF: Ciliary Neurotrophic Factor
DAPI: 4',6'-diamidino-2-phenylindole DNA stain
DS: Donkey Serum
GDNF: Glial cell line-Derived Neurotrophic Factor
GWAS: genome-wide association study
iPSC: Induced pluripotent stem cell
KHC: kinesin heavy chain
KIF5A: kinesin family member 5A
KLC: kinesin light chain
KO-DMEM:F12: KnockOut Dulbecco’s Modified Eagle Medium / Ham’s F-12
MN: motor neuron
NEAA: nonessential amino acid
NPC: neural progenitor cell
OPTN: Optineurin
PBMC: peripheral blood mononuclear cell
PBS: Phosphate Buffer Saline
RT: room temperature
RT-PCR: Reverse-transcriptase polymerase chain reaction
SAG dihydrochloride: Smoothened Agonist dihydrochloride
SETX: Senataxin
SFPQ: splicing factor proline- and glutamine-rich
SFPQ-RNA: Splicing Factor Proline- and Glutamine-Rich RNA-binding protein
SHH: Sonic Hedgehog, a recombinant signaling protein
SuperScript VILO: a reverse transcription (RT) kit sold by Thermo Fisher Scientific
TARDBP: TAR DNA-Binding Protein
TIAR: T-cell-restricted intracellular antigen-1 related protein
VCP: Valosin-Containing Protein
WGS: whole genome sequencing

## References

[1] Hardiman O, Al-Chalabi A, Chio A, et al. Amyotrophic lateral sclerosis. Nat Rev Dis Primers. 2017;3:17071. doi:10.1038/nrdp.2017.71 28980624

[2] Valdmanis PN, Rouleau GA. Genetics of familial amyotrophic lateral sclerosis. Neurology. 2008;70(2):144-152. doi:10.1212/01.wnl.0000296811.19811.db 18180444

[3] Rosen DR, Siddique T, Patterson D, et al. Mutations in Cu/Zn superoxide dismutase gene are associated with familial amyotrophic lateral sclerosis. Nature. 1993;362(6415):59-62. doi:10.1038/362059a0 8446170

[4] Mejzini R, Flynn LL, Pitout IL, Fletcher S, Wilton SD, Akkari PA. ALS genetics, mechanisms, and therapeutics: where are we now? Front Neurosci. 2019;13(Review):1310. doi:10.3389/fnins.2019.01310 31866818 PMC6909825

[5] Nicolas A, Kenna KP, Renton AE, et al. Genome-wide analyses identify KIF5A as a novel ALS gene. Neuron. 2018;97(6):1267-1288. doi:10.1016/j.neuron.2018.02.027 29566793 PMC5867896

[6] Brenner D, Yilmaz R, Müller K, et al. Hot-spot KIF5A mutations cause familial ALS. Brain. 2018;141(3):688-697. doi:10.1093/brain/awx370 29342275 PMC5837483

[7] Verhey KJ, Kaul N, Soppina V. Kinesin assembly and movement in cells. Annu Rev Biophys. 2011;40:267-288. doi:10.1146/annurev-biophys-042910-155310 21332353

[8] Brady ST. A novel brain ATPase with properties expected for the fast axonal transport motor. Nature. 1985;317(6032):73-75. doi:10.1038/317073a0 2412134

[9] Gindhart JG Jr., Desai CJ, Beushausen S, Zinn K, Goldstein LS. Kinesin light chains are essential for axonal transport in drosophila. J Cell Biol. 1998;141(2):443-454. doi:10.1083/jcb.141.2.443 9548722 PMC2148443

[10] Kanai Y, Okada Y, Tanaka Y, Harada A, Terada S, Hirokawa N. KIF5C, a novel neuronal kinesin enriched in motor neurons. J Neurosci. 2000;20(17):6374-6384. doi:10.1523/JNEUROSCI.20-17-06374.2000 10964943 PMC6772948

[11] Lim YW, James D, Huang J, Lee M. The emerging role of the RNA-binding protein SFPQ in neuronal function and neurodegeneration. Int J Mol Sci. 2020;21(19):7151. doi:10.3390/ijms21197151 32998269 PMC7582472

[12] Hogan AL, Grima N, Fifita JA, et al. Splicing factor proline and glutamine rich intron retention, reduced expression and aggregate formation are pathological features of amyotrophic lateral sclerosis. Neuropathol Appl Neurobiol. 2021;47(7):990-1003. doi:10.1111/nan.12749 34288034

[13] Widagdo J, Udagedara S, Bhembre N, et al. Familial ALS-associated SFPQ variants promote the formation of SFPQ cytoplasmic aggregates in primary neurons. Open Biol. 2022;12(9):220187. doi:10.1098/rsob.220187 36168806 PMC9516340

[14] Fukuda Y, Pazyra-Murphy MF, Silagi ES, et al. Binding and transport of SFPQ-RNA granules by KIF5A/KLC1 motors promotes axon survival. J Cell Biol. 2021;220(1):e202005051. doi:10.1083/jcb.202005051 33284322 PMC7721913

[15] Thomas-Jinu S, Gordon PM, Fielding T, et al. Non-nuclear pool of splicing factor SFPQ regulates axonal transcripts required for normal motor development. Neuron. 2017;94(4):931-336.e325. doi:10.1016/j.neuron.2017.04.036 28521142 PMC5441113

[16] van Rheenen W, van der Spek RAA, Bakker MK, et al. Common and rare variant association analyses in amyotrophic lateral sclerosis identify 15 risk loci with distinct genetic architectures and neuron-specific biology. Nat Genet. 2021;53(12):1636-1648. doi:10.1038/s41588-021-00973-1 34873335 PMC8648564

[17] Breevoort S, Gibson S, Figueroa K, Bromberg M, Pulst S. Expanding clinical spectrum of C9ORF72-related disorders and promising therapeutic strategies: a review. Neurol Genet. 2022;8(3):e670. doi:10.1212/NXG.0000000000000670 35620137 PMC9128039

[18] Bianchi F, Malboubi M, Li Y, et al. Rapid and efficient differentiation of functional motor neurons from human iPSC for neural injury modelling. Stem Cell Res. 2018;32:126-134. doi:10.1016/j.scr.2018.09.006 30278374

[19] Richards S, Aziz N, Bale S, et al. Standards and guidelines for the interpretation of sequence variants: a joint consensus recommendation of the American college of medical genetics and genomics and the association for molecular pathology. Genet Med. 2015;17(5):405-424. doi:10.1038/gim.2015.30 25741868 PMC4544753

[20] Nakano J, Chiba K, Niwa S. An ALS‐associated KIF5A mutant forms oligomers and aggregates and induces neuronal toxicity. Genes Cell. 2022;27(6):421-435. doi:10.1111/gtc.12936 PMC9322661 35430760

[21] Hasegawa M, Arai T, Nonaka T, et al. Phosphorylated TDP‐43 in frontotemporal lobar degeneration and amyotrophic lateral sclerosis. Ann Neurol. 2008;64(1):60-70. doi:10.1002/ana.21425 18546284 PMC2674108

[22] Lépine S, Nauleau-Javaudin A, Deneault E, et al. Homozygous ALS-linked mutations in TARDBP/TDP-43 lead to hypoactivity and synaptic abnormalities in human iPSC-derived motor neurons. iScience. 2024;27(3):109166. doi:10.1016/j.isci.2024.109166 38433895 PMC10905001

[23] Ratti A, Gumina V, Lenzi P, et al. Chronic stress induces formation of stress granules and pathological TDP-43 aggregates in human ALS fibroblasts and iPSC-motoneurons. Neurobiol Dis. 2020;145:105051. doi:10.1016/j.nbd.2020.105051 32827688

[24] Orrù S, Coni P, Floris A, et al. Reduced stress granule formation and cell death in fibroblasts with the A382T mutation of TARDBP gene: evidence for loss of TDP-43 nuclear function. Hum Mol Genet. 2016;25(20):4473-4483. doi:10.1093/hmg/ddw276 28172957

[25] Lenzi J, De Santis R, de Turris V, et al. ALS mutant FUS proteins are recruited into stress granules in induced pluripotent stem cell-derived motoneurons. Dis Model Mech. 2015;8(7):755-766. doi:10.1242/dmm.020099 26035390 PMC4486861

[26] Sidibé H, Khalfallah Y, Xiao S, et al. TDP-43 stabilizes G3BP1 mRNA: relevance to amyotrophic lateral sclerosis/frontotemporal dementia. Brain. 2021;144(11):3461-3476. doi:10.1093/brain/awab217 34115105 PMC8677511

[27] Udine E, Jain A, van Blitterswijk M. Advances in sequencing technologies for amyotrophic lateral sclerosis research. Mol Neurodegener. 2023;18(1):4. doi:10.1186/s13024-022-00593-1 36635726 PMC9838075

[28] Saez-Atienzar S, Dalgard CL, Ding J, et al. Identification of a pathogenic intronic KIF5A mutation in an ALS-FTD kindred. Neurology. 2020;95(22):1015-1018. doi:10.1212/WNL.0000000000011064 33077544 PMC7734922

[29] Zhang K, Liu Q, Shen D, et al. Mutation analysis of KIF5A in Chinese amyotrophic lateral sclerosis patients. Neurobiol Aging. 2019;73:229.e1-229.e4. doi:10.1016/j.neurobiolaging.2018.08.006 30301576

[30] Naruse H, Ishiura H, Mitsui J, et al. Splice-site mutations in KIF5A in the Japanese case series of amyotrophic lateral sclerosis. Neurogeneticsl 2021;22(1):11-17. doi:10.1007/s10048-020-00626-1 32815063

[31] Douglas AG, Baralle D. Reduced penetrance of gene variants causing amyotrophic lateral sclerosis. J Med Genet. 2024;61(3):294-297. doi:10.1136/jmg-2023-109580 38123999

[32] Xia CH, Roberts EA, Her LS, et al. Abnormal neurofilament transport caused by targeted disruption of neuronal kinesin heavy chain KIF5A. J Cell Biol 2003;161(1):55-66. doi:10.1083/jcb.200301026 12682084 PMC2172877

[33] Pant DC, Parameswaran J, Rao L, et al. ALS-linked KIF5A ΔExon27 mutant causes neuronal toxicity through gain-of-function. EMBO Rep. 2022;23(8):e54234. doi:10.15252/embr.202154234 35735139 PMC9346498

[34] Cozzi M, Magri S, Tedesco B, et al. Altered molecular and cellular mechanisms in KIF5A-associated neurodegenerative or neurodevelopmental disorders. Cell Death Dis. 2024;15(9):692. doi:10.1038/s41419-024-07096-5 39333504 PMC11437142

[35] Baron DM, Fenton AR, Saez-Atienzar S, et al. ALS-associated KIF5A mutations abolish autoinhibition resulting in a toxic gain of function. Cell Rep. 2022;39(1):110598. doi:10.1016/j.celrep.2022.110598 35385738 PMC9134378

[36] Campbell PD, Shen K, Sapio MR, Glenn TD, Talbot WS, Marlow FL. Unique function of kinesin Kif5A in localization of mitochondria in axons. J Neurosci. 2014;34(44):14717-14732. doi:10.1523/JNEUROSCI.2770-14.2014 25355224 PMC4212069

[37] Soustelle L, Aimond F, López-Andrés C, Brugioti V, Raoul C, Layalle S. ALS-associated KIF5A mutation causes locomotor deficits associated with cytoplasmic inclusions, alterations of neuromuscular junctions, and motor neuron loss. J Neurosci 2023;43(47):8058-8072. doi:10.1523/JNEUROSCI.0562-23.2023 37748861 PMC10669773

[38] Hackney DD, Baek N, Snyder AC. Half-site inhibition of dimeric kinesin head domains by monomeric tail domains. Biochemistry. 2009;48(15):3448-3456. doi:10.1021/bi8022575 19320433 PMC3321547

[39] Kaan HY, Hackney DD, Kozielski F. The structure of the kinesin-1 motor-tail complex reveals the mechanism of autoinhibition. Science. 2011;333(6044):883-885. doi:10.1126/science.1204824 21836017 PMC3339660

[40] Thomas-Jinu S, Gordon PM, Fielding T, et al. Non-nuclear pool of splicing factor SFPQ regulates axonal transcripts required for normal motor development. Neuron. 2017;94(4):931-336. e325. doi:10.1016/j.neuron.2017.04.036 28521142 PMC5441113

[41] Crimella C, Baschirotto C, Arnoldi A, et al. Mutations in the motor and stalk domains of KIF5A in spastic paraplegia type 10 and in axonal charcot-marie-tooth type 2. Clin Genet. 2012;82(2):157-164. doi:10.1111/j.1399-0004.2011.01717.x 21623771

[42] Chiba K, Ori-McKenney KM, Niwa S, McKenney RJ. Synergistic autoinhibition and activation mechanisms control kinesin-1 motor activity. Cell Rep. 2022;39(13):111016. doi:10.1016/j.celrep.2022.111016 35767955 PMC9475850

[43] Duranti E, Villa C. Molecular investigations of protein aggregation in the pathogenesis of amyotrophic lateral sclerosis. Int J Mol Sci. 2022;24(1):704. doi:10.3390/ijms24010704 36614144 PMC9820914

